# Supplementary material for: A generalized Flory-Stockmayer kinetic theory of connectivity percolation and rigidity percolation of cytoskeletal networks
Source: PLoS Comput Biol. 2022 May 9;18(5):e1010105. doi: 10.1371/journal.pcbi.1010105 (PMC9119625; doi:10.1371/journal.pcbi.1010105)
Supplement: S1 Text — Detailed description of the methods used for the chemical kinetic model, the generalization of the Flory-Stockmayer theory, and the coarse-grained mechanochemical model. (PDF) [file pcbi.1010105.s001.pdf]

Supplementary information for

A generalized Flory-Stockmayer kinetic theory of connectivity percolation and rigidity percolation of branched cytoskeletal networks

Carlos Bueno, James Liman, Nicholas P. Schafer, Margaret S. Cheung, and Peter G. Wolynes

I. Methods

A. Mass action chemical kinetics model

We quantified the concentration of the F-actin interfaces and their states (bound or unbound) using a mass action chemical kinetics model. Each F-actin monomer has 3 interfaces: the plus site (F_p), the minus site (F_m), and the binding site (F_c). The change of the concentration of plus sites bound to minus sites over time ($\frac{d[F_p \cdot F_m]}{dt}$) is given by the rate of polymerization at both ends, from which is subtracted the rate of depolymerization at both ends (Equation A). The polymerization rate on the plus end is proportional to the G-actin concentration ([G]), the concentration of unbound plus sites ($[F_p]$), and the rate constant for the polymerization on the plus end (k_p^+). The polymerization rate on the minus end is also proportional to the G-actin concentration ([G]), the concentration of unbound minus sites ($[F_m]$), and the rate constant for the polymerization on the minus end (k_m^+). The depolymerization rates do not depend on the G-actin concentration ([G]), only on the concentration of unbound plus or minus sites and on the respective rate constant (k_p^-, k_m^-). Free G-actin monomers ([G]) are consumed during the polymerization, so the change of G-actin concentration over time is the negative of the change of the concentration of plus sites bound to minus sites over time.

$$\frac{d[F_p \cdot F_m]}{dt} = -\frac{d[G]}{dt} = k_p^+[F_p][G] + k_m^+[F_m][G] - k_p^-[F_p] - k_m^-[F_m] \qquad \text{Equation A}$$

The change of the concentration of unbound plus sites over time ($\frac{d[F_p]}{dt}$) is given by the rate of creation of new filaments by branching, from which is subtracted the rate of destruction by unbranching (Equation B). The rate of creation of new filaments by branching is proportional to the concentration of unbound binding sites ($[F_c]$), the concentration of unbound minus sites ($[F_m]$), the concentration of unbound branchers ([B]), and the brancher binding rate constant (k_B^+). The rate of destruction of filaments by unbranching is proportional to the concentration of bound branchers ($[F_c \cdot B \cdot F_m]$), and the brancher unbinding rate constant (k_B^-).

$$\frac{d[F_p]}{dt} = k_B^+[F_c][G][B] - k_B^-[F_c \cdot B \cdot F_m] \qquad \text{Equation B}$$

On the other hand, since the branching reaction does not create new unbound minus sites (F_m), the concentration of unbound minus sites ($[F_m]$) is constant over time (Equation C). We did not include the actin filament nucleation or destruction processes in the chemical kinetics model.

$$\frac{d[F_m]}{dt} = 0 \qquad \text{Equation C}$$

The change of the concentration of bound branchers over time ($\frac{d[F_c \cdot B \cdot F_m]}{dt}$) is proportional to the creation rate of filaments by branching and is also the negative of the change in brancher concentration over time ($\frac{d[B]}{dt}$) (Equation D).

$$\frac{d[F_c \cdot B \cdot F_m]}{dt} = -\frac{d[B]}{dt} = k_B^+[F_c][G][B] - k_B^-[F_c \cdot B \cdot F_m] \qquad \text{Equation D}$$

Unless stated otherwise, the linker binding reaction occurs in a single step and the change in unbound linker concentrations over time ($\frac{d[L]}{dt}$) is given by the linker unbinding rate, from which is subtracted the linker binding rate. The linker unbinding rate is proportional to the concentration of bound linkers ($[c \cdot L \cdot c]$) and the linker unbinding rate constant (k_c^-). The linker binding rate is proportional to the concentration of unbound linkers ($[L]$), the square of the concentration of free binding sites ($[F_c]$), the linker binding rate constant (k_c^+,) and the spatial factor for the linker binding reaction (α_L). The change of the concentration of bound linker over time ($\frac{d[c \cdot L \cdot c]}{dt}$) is the negative of the concentration of unbound linkers (Equation E).

$$\frac{d[L]}{dt} = -\frac{d[c \cdot L \cdot c]}{dt} = -\alpha_L k_C^+[F_c]^2[L] + k_C^-[c \cdot L \cdot c] \qquad \text{Equation E}$$

The change of the unbound motor concentration over time ($\frac{d[M]}{dt}$) is given by the motor unbinding rate, subtracted by the motor binding rate. The motor unbinding rate is proportional to

the concentration of bound motors ([c · M · c]) and the motor unbinding rate constant (k_M^-). The motor binding rate is proportional to the concentration of unbound motors ([M]), the square of the concentration of free binding sites ($[F_c]$), the motor binding rate constant (k_M^+), and the spatial factor for the motor binding reaction (α_M). The change of the concentration of bound motor over time ($\frac{d[c \cdot M \cdot c]}{dt}$) is the negative of the concentration of unbound motors (Equation F).

$$\frac{d[M]}{dt} = -\frac{d[c \cdot M \cdot c]}{dt} = -\alpha_M k_M^+ [F_c]^2 [M] + k_M^- [c \cdot M \cdot c] \qquad \text{Equation F}$$

Finally, the change of the concentration of free binding sites over time ($\frac{d[F_c]}{dt}$) increases due to actin polymerization and decreases when the motor, linker, or brancher binds to a binding site (Equation G). A single free binding site becomes bound during the branching reaction, while two free actin binding sites become bound during the linker and motor binding reactions.

$$\begin{aligned} \frac{d[F_c]}{dt} = {} & \chi k_p^+ [F_p][G] - \chi k_p^- [F_p] + \chi k_m^+ [F_m][G] - \chi k_m^- [F_m] \\ & -k_B^+ [F_c][G][B] + k_B^- [F_c \cdot B \cdot F_m] \\ & -2\alpha_C k_C^+ [F_c]^2 [L] + 2k_C^- [F_c \cdot L \cdot F_c] \\ & -2\alpha_M k_M^+ [F_c]^2 [M] + 2k_M^- [F_c \cdot M \cdot F_c] \end{aligned} \qquad \text{Equation G}$$

This set of ordinary differential equations is based on the reactions of the Mechanochemical Dynamics of Active Networks (MEDYAN) model [1–6], developed by Papoian and his group (see section E of the supplementary information for more details). To make our set of equations comparable with the kinetic scheme of the MEDYAN model, we made the estimate that there is only one binding site for every 10 monomers, so unless stated otherwise $\chi = 0.1$. The spatial factor is defined as the probability that two actin binding sites are within the search distance in a homogeneous mixture.

Table A. Parameters used in the chemical kinetic model

Description	Constant	Value	Units
Polymerization rate coefficient on the plus end	k_p^+	11.6	$\mu M^{-1} s^{-1}$
Depolymerization rate coefficient on the plus end	k_p^-	1.4	s^{-1}
Polymerization rate coefficient on the minus end	k_m^+	1.3	$\mu M^{-1} s^{-1}$
Depolymerization rate coefficient on the plus end	k_m^-	0.8	s^{-1}
Linker binding rate coefficient	k_C^+	0.7	$\mu M^{-1} s^{-1}$
Linker unbinding rate coefficient	k_C^-	0.3	s^{-1}
Motor binding rate coefficient	k_M^+	0.7	$\mu M^{-1} s^{-1}$
Motor unbinding rate coefficient	k_M^-	1.7	s^{-1}
Brancher binding rate coefficient	k_B^+	0.0001	$\mu M^{-2} s^{-1}$
Brancher unbinding rate coefficient	k_B^-	1×10^{-10}	s^{-1}
Linker minimum search distance	d_C^{min}	30	nm
Linker maximum search distance	d_C^{max}	40	nm
Motor minimum search distance	d_M^{min}	175	nm
Motor maximum search distance	d_M^{max}	225	nm

B. The two-step model for the linker binding reaction

The affinity of the actin-binding proteins to single filaments has been shown to be important for domain separation of crosslinkers in experiments [7,8]. To reflect these experimental findings, we studied an alternative chemical kinetic model for linker binding. In this alternative model, each head of the linker binds an actin filament independently with the same binding affinity without cooperativity. We only included the polymerization, the depolymerization reactions, the linker binding, and the unbinding reactions (Table B).

Table B. Reactions included in the two-step chemical kinetic model for linker binding

REACTION	DESCRIPTION
$\boldsymbol{F_p + G \underset{k_p^-}{\overset{k_p^+}{\rightleftarrows}} F_p \cdot F_m + F_p + F_c}$	Actin polymerization and depolymerization at plus end
$\boldsymbol{F_m + G \underset{k_m^-}{\overset{k_m^+}{\rightleftarrows}} F_p \cdot F_m + F_m + F_c}$	Actin polymerization and depolymerization at minus end
$\boldsymbol{F_c + L \underset{k_C^-}{\overset{2k_C^+}{\rightleftarrows}} F_c \cdot L}$	Linker binding and unbinding
$\boldsymbol{F_c \cdot L + F_c \underset{k_C^-}{\overset{k_C^+}{\rightleftarrows}} F_c \cdot L \cdot F_c}$	Crosslinker formation and dissociation

In this model, the concentrations of the unbound plus sites (F_p) , the unbound minus sites (F_m), the plus sites bound to minus sites ($F_p.F_m$), and unbound G-actin (G) follow the Equation C, Equation A, and Equation B respectively assuming there is no brancher present. The concentration of free binding sites (F_c) is shown in Equation H.

$$\frac{d[F_c]}{dt} = -2\, k_c^+[F_c][L] - k_c^+[F_c \cdot L][F_c] + k_c^-[F_c \cdot L] + k_c^-[F_c \cdot L \cdot F_c] + k_p^+[F_p][G] + k_m^+[F_m][G] - k_p^-[F_p] - k_m^-[F_m] \quad \text{Equation H}$$

The concentration of linkers in the different states: unbound (L), single bound ($F_c \cdot L$), and double bound ($F_c \cdot L \cdot F_c$), are shown in Equation I, Equation J, and Equation K respectively.

$$\frac{d[L]}{dt} = -2\, k_c^+[F_c][L] + k_c^-[F_c \cdot L] \quad \text{Equation I}$$

$$\frac{d[F_c \cdot L]}{dt} = 2\, k_c^+[F_c][L] - k_c^+[F_c \cdot L][F_c] - k_c^-[F_c \cdot L] + k_c^-[F_c \cdot L \cdot F_c] \quad \text{Equation J}$$

$$\frac{d[F_c \cdot L \cdot F_c]}{dt} = k_c^+[F_c \cdot L][F_c] - k_c^-[F_c \cdot L \cdot F_c] \quad \text{Equation K}$$

C. Probability that an F-actin monomer is not connected to an infinite cluster (Ps)

The probability that an F-actin monomer is not connected to an infinite cluster through the site α (Q_α), is the sum of two terms. The first term is the probability of the site not being connected to another site ($1 - \theta_\alpha$). The second term is the probability that the site is connected to another F-actin monomer, with the condition that the neighboring bound F-actin monomer is not connected to an infinite cluster (Equation L).

$$Q_\alpha = 1 - \theta_\alpha + \sum_\beta \frac{\theta_{\alpha\to\beta}}{Q_\beta} \prod_\gamma Q_\gamma \quad \text{Equation L}$$

In this equation $\theta_\alpha = \sum_\beta \theta_{\alpha\to\beta}$, and α, β, and γ can be the plus site (p), the minus site (m), or the actin binding site (c). The probability that an F-actin monomer is not connected to an infinite cluster through any site (P_s) is the product of the probabilities of the F-actin monomer not being bound to an infinite cluster from each site (Q_α). (Equation M).

$$P_s = \prod_\alpha Q_\alpha \quad \text{Equation M}$$

In this equation α can be the plus site (p), the minus site (m), or the binding site (c). The system of equations given by Equation M has a trivial solution when $Q_p = Q_m = Q_c = 1$, which is the only solution when P_s=1 and the system is not percolated. This system can be solved exactly to calculate the fraction of actin monomers in finite clusters given the connectivity probabilities (θ_α) (Figure A).

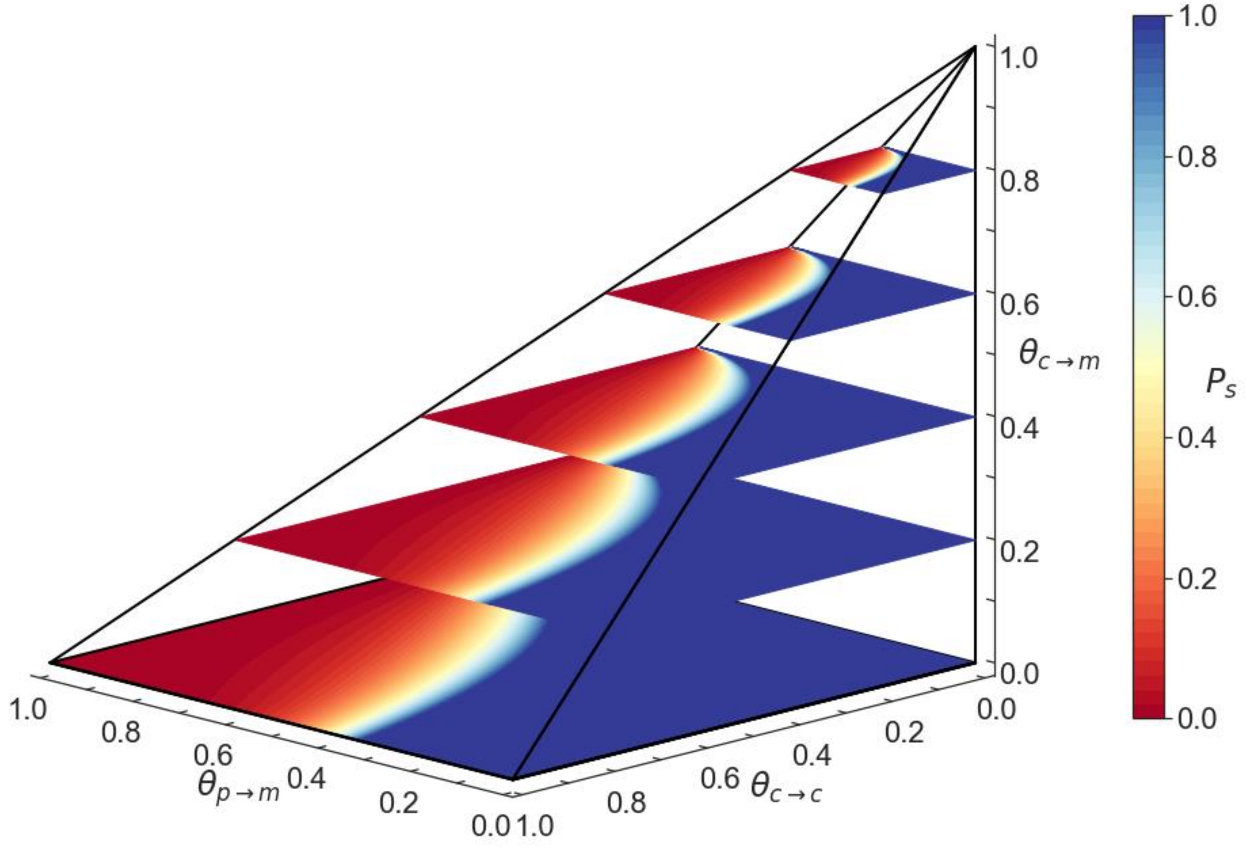

Figure A. Fraction of actin monomers in finite clusters (P_s) as a function of the crosslinking probabilities between plus and minus sites ($\theta_{p\rightarrow m}$), binding sites ($\theta_{c\rightarrow c}$), and binding sites to minus sites ($\theta_{c\rightarrow m}$). The color indicates the probability that an F-actin monomer is in a finite cluster.

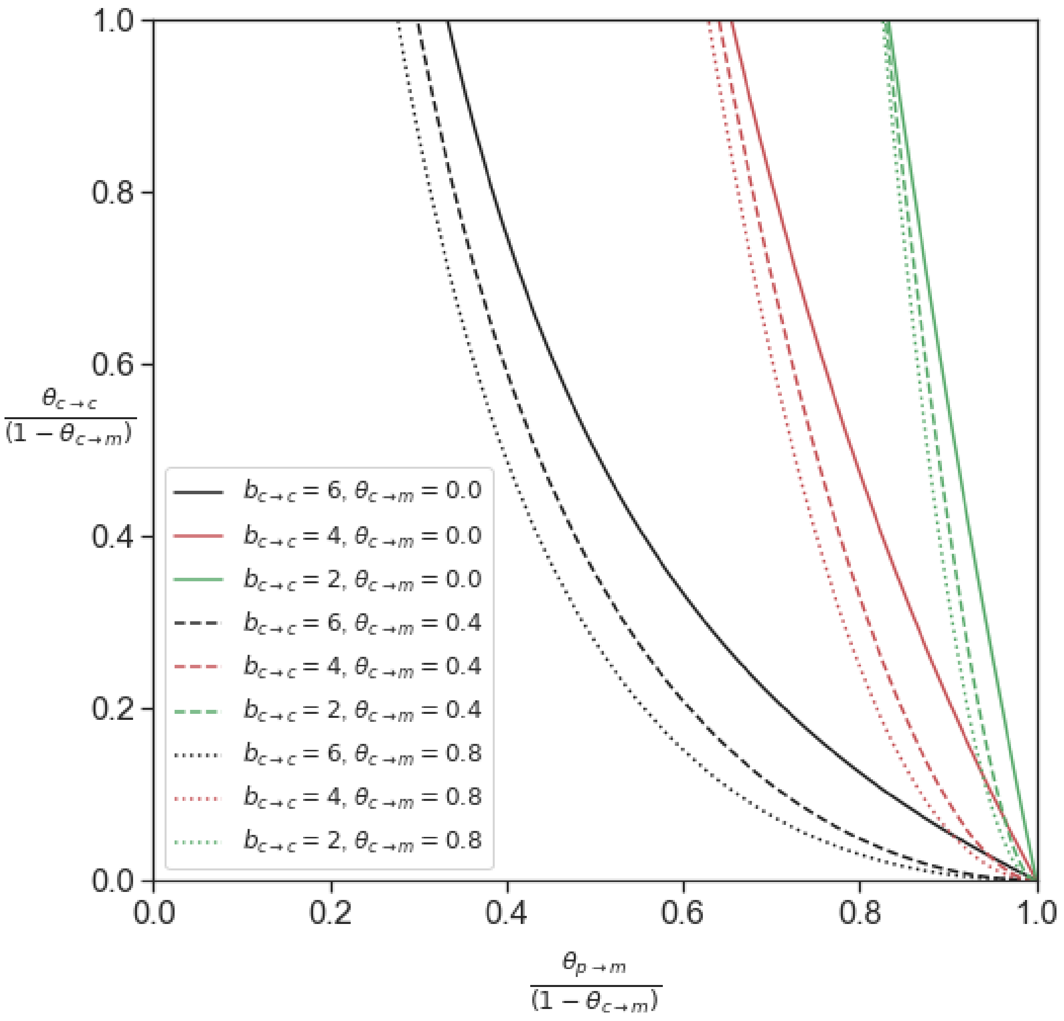

Figure B. Rigidity percolation boundaries as a function of the crosslinking probabilities between plus and minus sites ($\theta_{p\to m}$), binding sites ($\theta_{c\to c}$), binding sites and minus sites ($\theta_{c\to m}$), and the rigidity of the crosslinkers ($b_{c\to c}$). We assume that the connections between plus and minus sites, as well as the connections between binding sites and minus sites are rigid ($b_{p\to m} = b_{c\to m} = 6$)

D. Analytical solution of the Flory-Stockmayer equations for a system without branchers

We solved analytically the Flory-Stockmayer equations for a system where there is only actin and bivalent crosslinkers, like α-actinin or filamin. For this system, we calculated the

minimum concentration of linkers needed to percolate the system as a function of the connectivity probabilities ($\theta_{\alpha\to\beta}$). The probability of an actin monomer to be in a finite cluster is calculated by $P_s = \prod_\alpha Q_\alpha$, as shown in Equation M, where α can be the plus site (p), the minus site (m) or a binding site (c). Q is the probability that a site is not connected to an infinite cluster, as shown in Equation L. The analytical solution for Q for this system can be expressed by the following set of equations:

$$Q_p = 1 - \theta_{p\to m} + \theta_{p\to m} Q_p Q_c$$

$$Q_m = 1 - \theta_{m\to p} + \theta_{m\to p} Q_m Q_c$$

$$Q_c = 1 - \theta_{c\to c} + \theta_{c\to c} Q_p Q_m$$

In this system all connected minus sites are bound to plus sites. Then $\theta_p = \theta_{p\to m} = \theta_{m\to p} = \theta_m = \theta_a$, where we define θ_a as the probability of an F-actin minus site or plus site to be bound. We also define $\theta_c = \theta_{c\to c}$ as the probability that an F-actin binding site is bound. The previous system of equations can be reduced to the following set of equations.

$$Q_p = 1 - \theta_a + \theta_a Q_p Q_c$$

$$Q_m = 1 - \theta_a + \theta_a Q_m Q_c$$

$$Q_c = 1 - \theta_c + \theta_c Q_p Q_m$$

This set of equations has two solutions: a trivial solution $Q_c = Q_p = Q_m = 1$, and a non-trivial solution as shown below.

$$Q_c = \frac{1}{\theta_a} - \frac{\theta_c}{2} - \sqrt{\frac{\theta_c\,(4 - 4\theta_a + \theta_a\theta_c)}{4\theta_a}}$$

$$Q_p = Q_m = \sqrt{\frac{(4 - 4\theta_a + \theta_a\theta_c)}{4\theta_a\theta_c}} - \frac{1}{2}$$

The system percolates when $P_s = \prod_\alpha Q_\alpha < 1$, which occurs when:

$$\theta_c = \frac{1-\theta_a}{2\theta_a}$$

The average length of the filament ($\langle L \rangle$) is related to θ_a by the following equation:

$$\langle L \rangle = \frac{1}{1-\theta_a}$$

This equation shows that the percolation of the actomyosin network requires crosslinking. If the actin does not polymerize ($\theta_a = 0$), all F-actin monomers need to be crosslinked for the system to be percolated ($\theta_c = 1$). As the degree of polymerization increases, the length of the filament decreases and less crosslinkers are required to percolate the system. The proportion of crosslinkers required to percolate the network decreases hyperbolically as the length of the filament increases (Figure C).

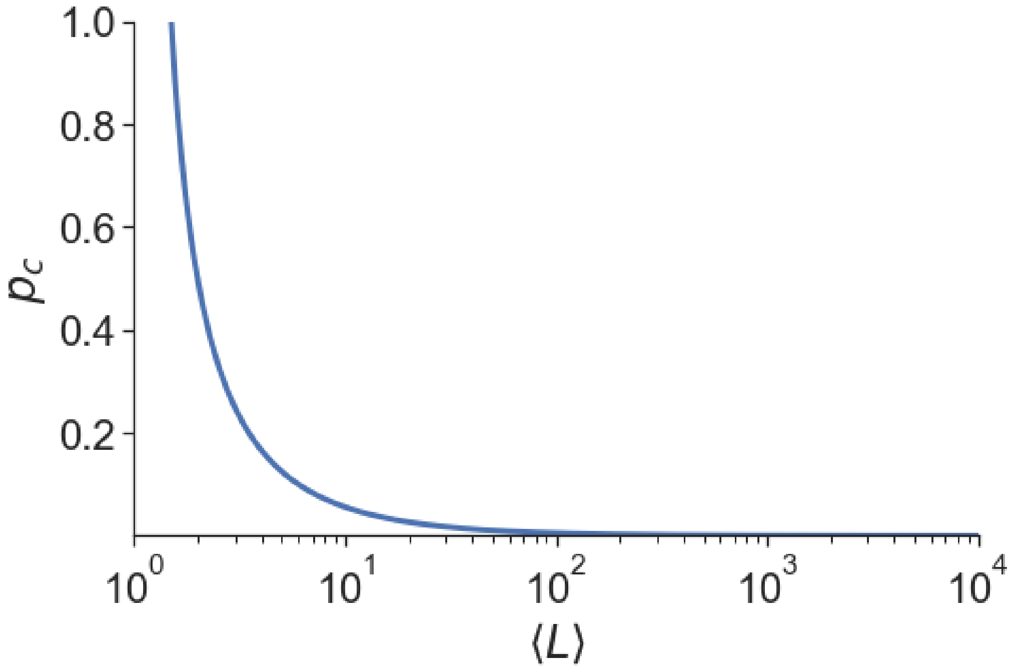

Figure C. Minimum crosslinking probability (θ_c) needed to percolate a system containing only linkers as a function of the average filament length ($\langle L \rangle$)

E. Coarse-grained mechanochemical model of actomyosin systems (MEDYAN)

We have used a coarse-grained mechanochemical model of actomyosin systems called MEDYAN (Mechanochemical Dynamics of Active Networks) developed by Papoian and his group [1–4,6]. MEDYAN models both stochastic chemical reactions and deterministic mechanical representations of far-from-equilibrium systems. In this study, we have included some important actin-binding proteins in actomyosin networks: non-muscle myosin IIA heavy chain (NMIIA) motors, α-actinin linkers, and actin-related protein complex 2/3 (Arp2/3) branchers, all in a fixed geometry.

MEDYAN represents actin filaments, linkers, motors, and branchers mechanically. For example, the actin filaments in MEDYAN are modeled as stretchable and bendable rods that have repulsive interactions with other filaments and the boundary. In our simulations a motor mini filament is an ensemble of, on average, 22.5 NMIIA subunits. MEDYAN simulations model chemical reactions stochastically using reaction-diffusion equations. Our MEDYAN simulations employ eight different compartments therefore allowing us to consider heterogeneous distributions of chemical species. Lastly, MEDYAN simulations consider mechanochemical feedback between the mechanical representations and the chemical reactions in the system. For example, the unbinding reaction of motors is modeled using a catch bond; hence motors are more likely to stay bound to a filament when pulling forces are applied to the motor.

We simulated a system containing 15000 actin monomers, 300 branchers, 333 motor mini-filaments, and 1500 linkers in a 1μm^3 cubic box, which corresponds to an actin concentration of 25μM, a linker concentration of 2.5 μM, a motor concentration of 12.5 μM, and a brancher concentration of 0.5 μM. The branchers were introduced after 1 second. The parameters for the simulations are the same as the ones shown in Table A. The simulations were run with and without

branchers. Sample snapshots of the simulation are shown in Figure D. We followed the same procedure as described by in our previous work [5]. We compared the concentration of bound species to the concentration of bound species found in the chemical kinetic model. To compare the discrete snapshots of MEDYAN to the continuum chemical kinetic model, we ran 5 different simulations with different snapshot intervals, each 0.001s, 0.01s, 0.1s, 1s, and 10s, and interpolated the obtained data using a linear interpolation with the closest snapshots to obtain a mean MEDYAN concentration. G-actin monomers, F-actin filaments, α-actinin linkers, NMIIA motors, and Arp2/3 branchers were initially distributed randomly inside a cubic container. All MEDYAN simulations were enclosed in a $1\ \mu m \times 1\ \mu m \times 1\ \mu m$ container with 25 μM of actin in total as described in our previous work [5].

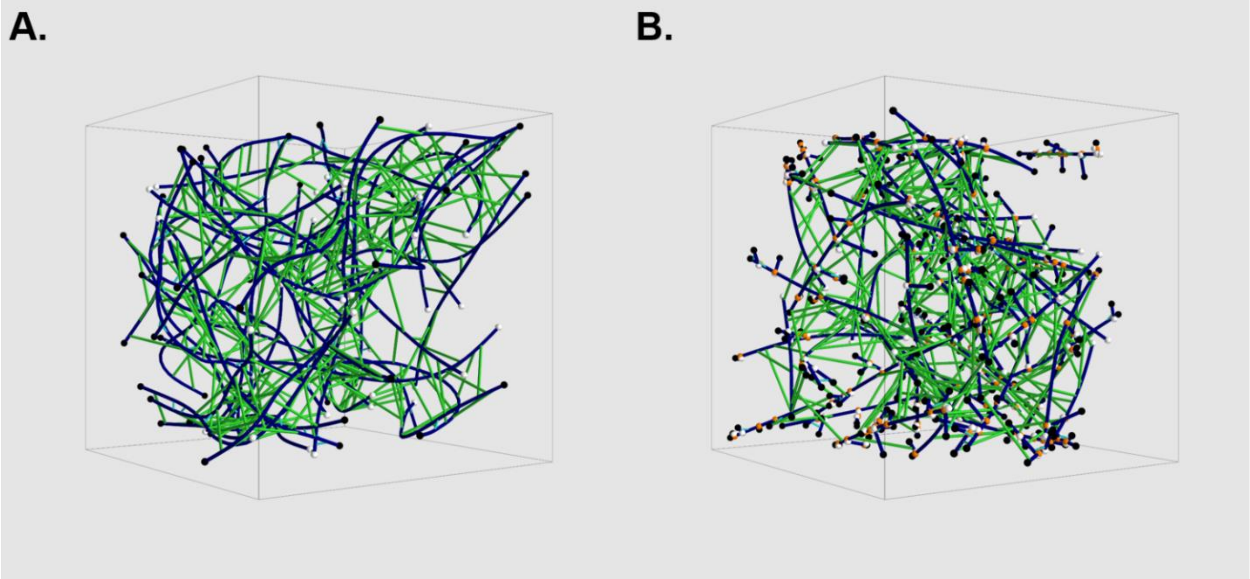

Figure D. Typical snapshot of MEDYAN simulation of an unbranched network simulation (A) and a branched network simulation (B). Blue cylinders represent actin filaments, green cylinders represent motor mini-filaments, cyan cylinders represent crosslinkers, white beads represent the minus end, black beads represent the plus end, and orange beads represent branchers.

References

1. Popov K, Komianos J, Papoian GA. MEDYAN: Mechanochemical Simulations of Contraction and Polarity Alignment in Actomyosin Networks. PLoS Comput Biol. 2016;12:

1–35. doi:10.1371/journal.pcbi.1004877. PubMed PMID: 27120189

2. Ni Q, Papoian GA. Turnover versus treadmilling in actin network assembly and remodeling. Cytoskeleton (Hoboken). 2019;76: 562–570. doi:10.1002/cm.21564. PubMed PMID: 31525282

3. Floyd C, Papoian GA, Jarzynski C. Quantifying dissipation in actomyosin networks. Interface Focus. 2019;9: 20180078. doi:10.1098/rsfs.2018.0078. PubMed PMID: 31065344

4. Komianos JE, Papoian GA. Stochastic Ratcheting on a Funneled Energy Landscape Is Necessary for Highly Efficient Contractility of Actomyosin Force Dipoles. Phys Rev X. 2018;8: 21006. doi:10.1103/PhysRevX.8.021006

5. Liman J, Bueno C, Eliaz Y, Schafer NP, Waxham MN, Wolynes PG, et al. The role of the Arp2/3 complex in shaping the dynamics and structures of branched actomyosin networks. Proc Natl Acad Sci U S A. 2020;117: 10825–10831. doi:10.1073/pnas.1922494117. PubMed PMID: 32354995

6. Chandrasekaran A, Upadhyaya A, Papoian GA. Remarkable structural transformations of actin bundles are driven by their initial polarity, motor activity, crosslinking, and filament treadmilling. PLoS Comput Biol. 2019;15: e1007156. doi:10.1371/journal.pcbi.1007156. PubMed PMID: 31287817

7. Freedman SL, Suarez C, Winkelman JD, Kovar DR, Voth GA, Dinner AR, et al. Mechanical and kinetic factors drive sorting of F-actin cross-linkers on bundles. Proc Natl Acad Sci U S A. 2019;116: 16192–16197. doi:10.1073/pnas.1820814116. PubMed PMID: 31346091

8. Winkelman JD, Suarez C, Hocky GM, Harker AJ, Morganthaler AN, Christensen JR, et al.

Fascin- and α-Actinin-Bundled Networks Contain Intrinsic Structural Features that Drive Protein Sorting. Curr Biol. 2016;26: 2697–2706. doi:10.1016/j.cub.2016.07.080. PubMed PMID: 27666967
