# Supplementary material for: A generalized Flory-Stockmayer kinetic theory of connectivity percolation and rigidity percolation of cytoskeletal networks
Source: PLoS Comput Biol. 2022 May 9;18(5):e1010105. doi: 10.1371/journal.pcbi.1010105 (PMC9119625; doi:10.1371/journal.pcbi.1010105)
Supplement: Attachment — Submitted filename: FSReviewer_responsev4.pdf [file pcbi.1010105.s003.pdf]

Dear Editor and Reviewers,

Thank you for your thoughtful reviews. We have made several changes to clarify the points made. We detail them in the responses below. In addition, in recognition of their contributions, we have also added an acknowledgement to Dr. Neal Waxham and Dr. Garegin Papoian for their helpful discussions on this project.

"We thank both Neal Waxham and Garegin Papoian for their helpful discussions and acknowledge with gratitude the use of the MEDYAN code provided by Dr. Papoian."

Reviewer #1:

The authors have compared a newly developed kinetic theory for the growth of actomyosin networks with computer simulations in the software environment MEDYAN. The kinetic theory is a new version of the Flory-Stockmayer theory for gelation and similar to the one presented in Ref. 48 by Sciortino and coworkers for patchy particles with three interaction sites (JCP 2010).

Here the three different interaction sites for F-actin are minus end, plus end and a lateral binding site for linkers, branchers and motors. The comparison with MEDYAN shows that the kinetic theory works surprisingly well.

The big advantage is that it can be interrogated for questions that are not so easily answered in computer simulations and experiments, in particular for the percolation transition by Maxwell counting.

This work is a nice theory extension to the MEDYAN-simulations of branched networks published by the authors as Ref. 19 (PNAS 2020) and certainly deserves publication somewhere.

1) In my view, however, it would be much better suited for more theoretically oriented journals such as JCP (physical chemistry community) or PRE (statistical physics community). For PLOS Comp Biol, I am missing the direct biological relevance. As explained in the following, in my view there are several issues from the biophysics point of view.

Thank you for the insightful comments on our paper. We are interested in publishing this research in PLOS Computational Biology since while we agree the soft matter community would appreciate our findings, our results and ideas are also relevant for the biological audience who would appreciate the relevance of these physical concepts by seeing them implemented for a specific biological system of significant intricacy to be realistic. In fact, our work in this field is partially motivated by the dendritic spine, a specific biological system having many of the components we discussed as well as others. Publishing this work in journals such as JCP or PRE would raise questions in their audience of why we are choosing to model such complex systems. The reason why we chose to model actomyosin networks has been the behavior of dendritic spines in memory, but in general the theory presented here can be applied to many other complex biological systems.

There have been many papers that have cited the role of connectivity in simplified models, such as Ennomani et al. and MacKintosh et al. We believe that the Flory-Stockmayer theory however gives a novel insight into the mechanism by which the connectivity actually is established in actomyosin systems.

Nevertheless, our model remains a simplification of the actomyosin network which does lack some details relevant to mechanical properties such as the geometry, orientation of the actin filaments and actin binding proteins, steric effects, crowding, but we think the main themes relevant to establishing connectivity are well captured.

We emphasize that the intention of this paper is to present a simplified model of the interactions between actin-binding proteins and actin filaments highlights how the connectivity, rigidity, and force percolation phenomena are established by the kinetic growth of the cytoskeleton. In our system the filaments are dispersed isotropically, so we do not expect all motors to exert significant vectorial force. Overall, some of the motors do exert forces, which can only propagate once the system is connected, leading to isotropic contraction rather than vectorial flow. These simplifications lead to a simple soluble deterministic system which presents a clear percolation transition which is akin to the transitions actually observed in cells.

We added this sentence in line 59 of the main text:

“For example in neurons, the complex structure of actomyosin networks in the dendritic spines are regulated by actin-binding proteins such as non-muscle myosin IIA heavy chain (NMIIA) motors, α-actinin, actin-related protein complex 2/3 (Arp2/3), and calcium/calmodulin-dependent protein kinase II (CaMKII) [13,14].”

2) In the cell, it just does not happen that all described processes compete with each other at once. Most importantly, myosin needs bipolar actin to contract actin, but branched networks cannot be bipolar. A great experimental demonstration of the importance of bipolar actin for contraction was given by Manuel Thery and Laurent Blanchoin with micropatterning (compare Reymann, Anne-Cécile, et al. "Actin network architecture can determine myosin motor activity." Science 336.6086 (2012): 1310-1314 and Ennomani, Hajer, et al. "Architecture and connectivity govern actin network contractility." Current Biology 26.5 (2016): 616-626). This important subject is not discussed here and a reader not familiar with actomyosin might overlook that branched networks cannot contract.

In this model we considered only an overall disordered isotropic actin network. In such an unorganized actin network some myosin motors will be able to generate force when their attached actin filaments are antiparallel, while other myosin motors will only connect parts of the network. In this way, on average, some forces will be produced by the myosin motors in a disordered network leading to contraction as shown by Ennomani et al.

We have added the following sentence to the Results section:

“This discrepancy arises from the heterogeneous distribution of the binding sites in the system. In the chemical kinetic model, a homogeneous distribution of binding sites is assumed, while in MEDYAN the distribution eventually becomes spatially heterogeneous”

To this:

“This discrepancy arises from the heterogeneous distribution of the binding sites in the system. In the chemical kinetic model, a homogeneous distribution of binding sites and an isotropic network conformation is assumed, while in MEDYAN the distribution is spatially heterogeneous and the filaments can form bundles”

3)In general the authors focus most of their discussion on Arp2/3, but as far as I can see, the famous 70 degree angle also implemented in their PNAS-paper with MEDYAN is not used here. It is my understanding that this is be a severe limitation of the Stockmayer-like approach. On the other hand, this is an essential element of the biological system, for example because it has been shown that branched actin networks can undergo phase transitions between different architectures due to this 70 degree branching angle (compare Mueller, Jan, et al. "Load adaptation of lamellipodial actin networks." Cell 171.1 (2017): 188-200).

The reason the 70-degree angle is not included in the kinetics part of this paper is because we specifically selected to focus on understanding the network connectivity rather than the complete geometry of the system. We understand that the geometry of the system and the 70-degree angle detail can be important in establishing liquid crystallinity, etc. or when the actomyosin system interacts with the membrane. The angular constraint indeed is included in the MEDYAN simulations, but this does not affect greatly the connectivity at these concentrations.

4)I am also not sure how realistic is the representation of the motor elements. Single myosin II molecules do not contract actin networks, but myosin II minifilaments do. Minifilaments need bipolar actin and use dozens of motor heads to bind to it. This has been implemented in Cytosim (Cortes, Daniel B., et al. "Bond type and discretization of nonmuscle myosin II are critical for simulated contractile dynamics." Biophysical journal 118.11 (2020): 2703-2717), which is an alternative to MEDYAN, but it is not clear how this is being done here and how well this corresponds to experiments.

Thank you for mentioning this, which uncovers a possible misreading caused by our bad word choice, which we have clarified throughout the text. The motors both in MEDYAN simulations and in the chemical kinetic model have multiple heads, they are not single myosin molecules. In MEDYAN simulations the minifilaments have between 15 and 30 heads (selected using a uniform random distribution), while in the chemical kinetic model each myosin motor has 22.5 heads following this reference:

Chandrasekaran A, Upadhyaya A, Papoian GA. Remarkable structural transformations of actin bundles are driven by their initial polarity, motor activity, crosslinking, and filament treadmilling. PLoS Comput Biol. 2019;15: e1007156. doi:10.1371/journal.pcbi.1007156. PubMed PMID: 31287817

To clarify our terminology, we have added this sentence:

“To account for the NMIIA minifilaments, a motor (M) consists of 22.5 myosin molecules, which corresponds to the average number of motor heads in the MEDYAN model [7,19].”

We also found a typo in the following sentence:

“In muscle cells actin filaments and the accompanying myosin monofilaments are parallel and organized, and the mechanism of contraction is relatively well understood [2].”

Which has now been modified to read:

“In muscle cells actin filaments and the accompanying myosin minifilaments are parallel and organized, and the mechanism of contraction is relatively well understood [2].”

5) In my understanding, the clearest relations to experiment could be the predictions on percolation, but it is not explained if there are experimental data that back these up. Experiments are mentioned in passing in Fig. 4, but only in a negative manner (not relevant).

Our results correlate to the experimental results of Bendix et al. (2008), which show that the macroscopic contractility in actin reconstituted systems requires a threshold concentration of motors but is strongest with an intermediate concentration of linkers.

Bendix PM, Koenderink GH, Cuvelier D, Dogic Z, Koeleman BN, Brieher WM, et al. A Quantitative Analysis of Contractility in Active Cytoskeletal Protein Networks. Biophys J. 2008;94: 3126–3136. doi:10.1529/biophysj.107.117960. PubMed PMID: 18192374

This re-entrant phase is also observed in our theoretical model and was discussed earlier by S Wang and PG Wolynes in the following article:

Wang S, Wolynes PG. Active contractility in actomyosin networks. Proc Natl Acad Sci. 2012;109: 6446–6451. doi:10.1073/pnas.1204205109. PubMed PMID: 22493220

Based on the feedback we have modified our description of the experiments in Figure 4 from this sentence:

“A small survey of experiments in the literature showed that most of the concentrations at which the actin-binding proteins have been studied were less than the concentration threshold required to observe a decrease in the number of crosslinks as the total concentration of crosslinkers increases (Fig 4).”

This has now been modified to read:

“A small survey of experiments in the literature shows that the first connectivity percolation transition has been observed when the system is not saturated by linkers. (Fig 4).”

We also modified the figure and the caption from this:

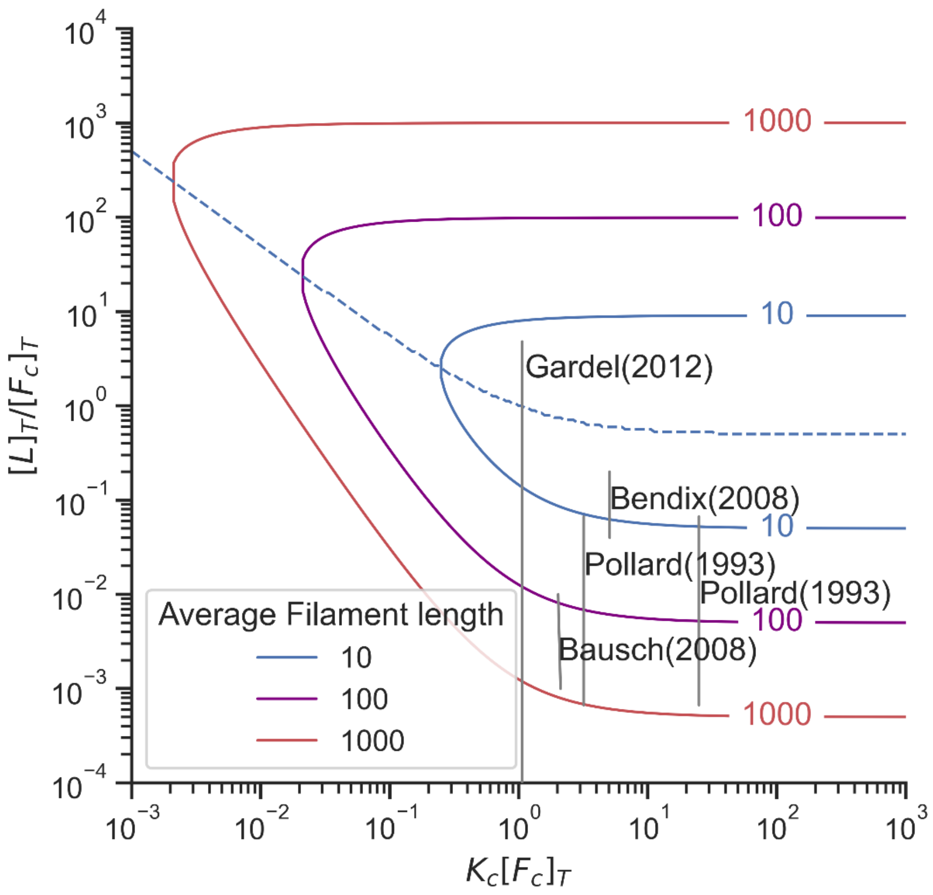

Fig 4. Plot showing the location of different experiments on actin crosslinking plotted in the two-step model phase space [5,11,58,59]. The lines indicate percolation transitions for filaments of different sizes. The dotted blue line indicates the maximum number of crosslinks. $[L]_T$ is the total linker concentration, $[Fc]_T$ is the total concentration of binding sites, and Kc is the linker binding equilibrium constant.

To this:

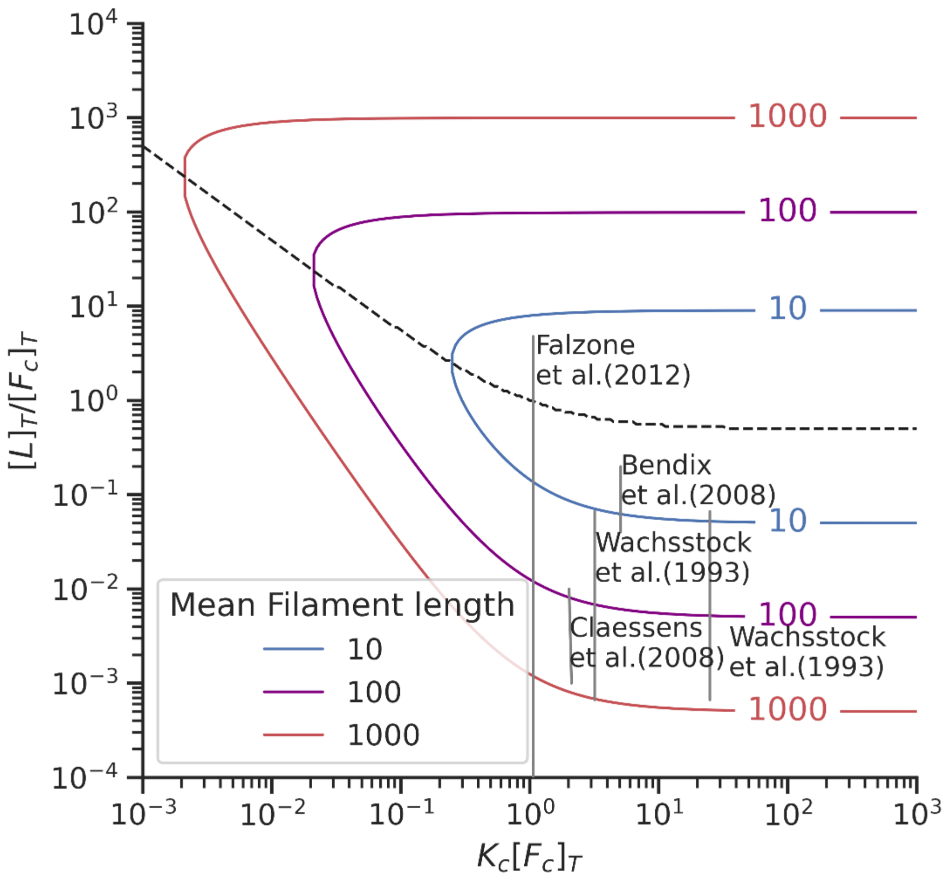

Fig 4. Plot showing the location of different experiments on actin crosslinking plotted in the two-step model phase space [5,11,58,59]. The curved lines indicate percolation transitions for filaments of different sizes. The dotted black line indicates the region where the maximum number of crosslinks can be observed. $[L]_T$ is the total linker concentration, $[Fc]_T$ is the total concentration of binding sites, and Kc is the linker binding equilibrium constant.

6) Apart from MEDYAN and Cytosim, one should also mention AFINES, as done in the PNAS-paper.

We have added a citation for the AFINES model.

The sentence,

"Several models have been developed to simulate interactions between actin-binding proteins and actin filaments such as MEDYAN [37] and Cytosim [38]"

has been modified to read,

"Several models have been developed to simulate interactions between actin-binding proteins and actin filaments such as MEDYAN [37], Cytosim [38], and AFINES [39]".

We also added a reference to AFINES.

“39. Freedman SL, Banerjee S, Hocky GM, Dinner AR. A Versatile Framework for Simulating the Dynamic Mechanical Structure of Cytoskeletal Networks. Biophys J. 2017;113: 448–460. doi:10.1016/j.bpj.2017.06.003. PubMed PMID: 28746855”

7) Line 145 typo: In the main the transient concentrations

The sentence,

“In the main the transient concentrations from the chemical kinetic model and MEDYAN simulations agree with each other as shown in Fig 1.”

has been modified to read,

“In the main, the transient concentrations from the chemical kinetic model and MEDYAN simulations agree with each other as shown in Fig 1.”

8) Line 356 typo: In our previous works [19], we modeled linker binding as a termolecular reaction

The sentence,

“In our previous works [19], we modeled linker binding as a termolecular reaction in which a linker simultaneously binds two actin filaments, forming a crosslink. Termolecular reactions can however be decomposed into two separate bimolecular steps.”

has been modified to read,

“In our previous works [19], we modeled linker binding as a termolecular reaction in which a linker must simultaneously bind two actin filaments, forming a crosslink. Termolecular reactions in biology can however be decomposed into two separate bimolecular steps.”

Reviewer #2:

The paper compares the calculated dynamic structure (and rigidity) of actomyosin networks under polymerization, association with actin binding proteins, branching, and active, myosin originated, forces. Two approaches are used. One is mass action chemical kinetics (i.e. mean-field kinetics), to obtain both for the dynamics and steady-state probabilities, that is combined with Maxwell approach for network mechanical stability (rigidity percolation). Henceforth I term this approach as mean-field. The other approach is the MEDYAN simulation package that was developed by Papoian and co-workers and accounts for the actual inhomogeneities that can appear during such out-of-equilibrium polymerization. It is gratifying that both approaches yield similar results below the percolation threshold, which basically demonstrates that the system remains homogeneous during polymerization and crosslinking. Above percolation threshold, the difference between the two approaches is attributed to heterogeneity. I believe this is a worthy paper, demonstrating that a simplified approach can also work for such an enormously complicated system.

Before entering into more details, I would like to address the issue of novelty. MEDYAN appears as powerful computational approach but it is not the main focus of this work. In fact, Papoian is not a co-author. The MEDYAN results are presented here as a confirmation of the mean-field results, along with some experimental results appearing in the literature. While I do appreciate a lot the mean-field

approach, I find it much less novel than MEDYAN, so it does make me wonder if this paper should appear in PLOS Computational Biology.

We have reached out to Dr. Garegin Papoian and after our discussion he indicated that he felt his contributions would not be enough for him to feel comfortable being a coauthor for this paper. MEDYAN is a simulation software that can be and has already been used for multiple applications. Our paper focuses specifically on explaining how the actin network connectivity is linked to contractility and uses MEDYAN results as a comparison with the kinetic theory, many of which already were published by us in another paper, a paper again that Dr. Papoian did not choose to co-author with us. We believe the present paper by itself presents novel insights distinct from what MEDYAN by itself provides.

We must add that we are happy to acknowledge the contributions of discussions with Dr. Neal Waxham and Dr. Garegin Papoian. We added the following sentence in acknowledgements:

"We thank both Neal Waxham and Garegin Papoian for their helpful discussions and acknowledge with gratitude the use of the MEDYAN code provided by Dr. Papoian."

I have a few more specific questions:

The paper mentions three phases (sol, gel, active). I am not sure what is the active phase. What prevents the motors to exert forces in in either the sol or gel phase? Surely I can believe there is positive coupling between motor activity and the resulting structure and vice-versa, e.g., motors can help forming a gel (acting as linkers) while also exerting forces that influence the binding of linkers. However, I do not see why myosin cannot exert forces on finite clusters in the sol phase, which may also propagate through the solvent and effect partially other finite clusters.

The reason why we describe the actin as being active only in the "active phase" is because in the sol or gel phases not enough connections are formed in the actin network to be able to transmit the forces in a global fashion. In the sol phase local contractions doubtlessly are observable in the system, but global contractions would not be observed since there are not enough connections in the system. Since motors can exert forces locally, but not globally, such a system in this regime would only appear active in small regions. On the other hand, once all the system becomes connected, but not too rigid, the system becomes able to contract globally, our meaning of "active"

We did not consider propagation of force through hydrodynamics which we consider outside of the scope of our simplified model.

We added the following sentence:

"Rigidity and force propagation through other mechanisms in the actin network, such as hydrodynamics, could also play a significant factor in contraction. These mechanisms are outside of the scope of this paper.

I would also like to enquire why no "aster phase" or "star phase" is seen (e.g.: https://www.pnas.org/content/pnas/103/13/4906.full.pdf). Can the authors suggest an explanation? I understand why it is not predicted by mean field, but what about MEDYAN? Is it an issue with the experiment or perhaps MEDYAN is too coarse grained?

Studying the aster phase was outside the objective of our paper since the kinetic model does not include the detailed geometry of the actin network. It is something that we are interested on working on the future as an extension of the model. We do note however that the aster phase has been studied using MEDYAN as reported in the following article:

Chandrasekaran A, Upadhyaya A, Papoian GA. Remarkable structural transformations of actin bundles are driven by their initial polarity, motor activity, crosslinking, and filament treadmilling. PLoS Comput Biol. 2019;15: e1007156. doi:10.1371/journal.pcbi.1007156. PubMed PMID: 31287817

I wonder about the actual role of filament elasticity, as a linear object, branched, or crosslinked. I did not see anywhere discussion on the effect of the elastic or viscoelastic response of the network. How do forces generated by motors propagate first through the individual filaments (that obey bending elasticity), and then through the branches and crosslinks (as well as other motors serving as crosslinks), possibly influencing the binding of other motors/linkers and the activity of other motors?

This is something that we have given a lot of thought and we also believe may be an important factor that limits the ability of Maxwell counting to account for the rheology by itself as discussed by Wang and Wolynes earlier. Strictly speaking the rigidity percolation phenomena as described by Thorpe et al. describes inflexible systems where the perfectly rigid interactions of the particles determine the number of degrees of freedom of the system. In our model the degrees of freedom of the crosslinker and motor connections may depend on whether the system is in an extended state, a contracted state, or a relaxed state. In this paper we do not formally address how many effective degrees of freedom remain in each state and limited ourselves to indicate what would happen in a completely flexible state or in a completely rigid state. We believe that actin linkers and motors behave in a manner between the flexible and rigid connections described in the paper and the entropy losses are not precisely determined by the number of degrees of freedom alone, but also by steric effects, etc. On the other hand, we have treated actin filaments as rigid objects, since they are rigid in comparison to crosslinkers or motors. Their flexibility may also play a role in rigidity and force percolation.

It is well established that myosin acts cooperatively. Do such effects are taken by MEDYAN?

Yes, there are cooperative effects present in MEDYAN due to the alignment of the filaments, which is caused by both motor walking and actin treadmilling. Also the model includes multiple heads binding the same filament and cooperatively increasing the binding time. This has been described in the following article:

Popov K, Komianos J, Papoian GA. MEDYAN: Mechanochemical Simulations of Contraction and Polarity Alignment in Actomyosin Networks. PLoS Comput Biol. 2016;12: 1–35. doi:10.1371/journal.pcbi.1004877. PubMed PMID: 27120189

Can the authors address how would a more accurate study of rigidity percolation, e.g., the study of Broedersz, MacKintosh and coworkers, would affect the results?

The rigidity percolation studies of Broedersz and MacKintosh are concerned with the elastic behavior of actin networks in regular 2D triangular lattices and in 3D fcc lattices which have been decimated by cutting bonds while our model is based on a Bethe-like lattice, which has been built by chemical association. We believe the actual lattice represents more realistically the connectivity of biological actin

networks than does a decimated fcc lattice and is the basis of the extension of the Flory-Stockmayer theory presented here.

To conclude, while the paper is interesting and could worthy publication, I do not recommend publication in its present form in PLOS Computational Biology. MEDYAN should be presented thoroughly, including Papoyan as a co-author, it should address the above questions, and include many more results showing the heterogeneities formed in the system. Otherwise, I think the paper does suit in its present form publication in a physical chemistry journal.

Avalanches and heterogeneities were featured in a major way in our previous PNAS paper on this system.

Liman J, Bueno C, Eliaz Y, Schafer NP, Waxham MN, Wolynes PG, et al. The role of the Arp2/3 complex in shaping the dynamics and structures of branched actomyosin networks. Proc Natl Acad Sci U S A. 2020;117: 10825–10831. doi:10.1073/pnas.1922494117. PubMed PMID: 32354995

They are not the focus of this paper which studies the adequacy of a spatially averaged kinetic model.

Reviewer #2: NO: MEDYAN code was not provided

The MEDYAN code is available at www.medyan.org. We have provided the files needed to run the simulation and the results at https://doi.org/10.5281/zenodo.5645714.
