# Supplementary material for: A generalized Flory-Stockmayer kinetic theory of connectivity percolation and rigidity percolation of cytoskeletal networks
Source: PLoS Comput Biol. 2022 May 9;18(5):e1010105. doi: 10.1371/journal.pcbi.1010105 (PMC9119625; doi:10.1371/journal.pcbi.1010105)
Supplement: Attachment — Submitted filename: referee-report-Plos-com-bio-19.1.docx [file pcbi.1010105.s002.docx]

The paper compares the calculated dynamic structure (and rigidity) of acto-myosin networks under polymerization, association with actin binding proteins, branching, and active, myosin originated, forces. Two approaches are used. One is mass action chemical kinetics (i.e. mean-field kinetics), to obtain both for the dynamics and steady-state probabilities, that is combined with Maxwell approach for network mechanical stability (rigidity percolation). Henceforth I term this approach as mean-field. The other approach is the MEDYAN simulation package that was developed by Papoian and co-workers and accounts for the actual inhomogeneities that can appear during such out-of-equilibrium polymerization. It is gratifying that both approaches yield similar results below the percolation threshold, which basically demonstrates that the system remains homogeneous during polymerization and crosslinking. Above percolation threshold, the difference between the two approaches is attributed to heterogeneity. I believe this is a worthy paper, demonstrating that a simplified approach can also work for such an enormously complicated system.

Before entering into more details, I would like to address the issue of novelty. MEDYAN appears as powerful computational approach but it is not the main focus of this work. In fact, Papoian is not a co-author. The MEDYAN results are presented here as a confirmation of the mean-field results, along with some experimental results appearing in the literature. While I do appreciate a lot the mean-field approach, I find it much less novel than MEDYAN, so it does make me wonder if this paper should appear in PLOS Computational Biology.

I have a few more specific questions:

1. The paper mentions three phases (sol, gel, active). I am not sure what is the active phase. What prevents the motors to exert forces in in either the sol or gel phase? Surely I can believe there is positive coupling between motor activity and the resulting structure and vice-versa, e.g., motors can help forming a gel (acting as linkers) while also exerting forces that influence the binding of linkers. However, I do not see why myosin cannot exert forces on finite clusters in the sol phase, which may also propagate through the solvent and effect partially other finite clusters.
2. I would also like to enquire why no "aster phase" or "star phase" is seen (e.g.: <https://www.pnas.org/content/pnas/103/13/4906.full.pdf> ). Can the authors suggest an explanation? I understand why it is not predicted by mean field, but what about MEDYAN? Is it an issue with the experiment or perhaps MEDYAN is too coarse grained?
3. I wonder about the actual role of filament elasticity, as a linear object, branched, or crosslinked. I did not see anywhere discussion on the effect of the elastic or viscoelastic response of the network. How do forces generated by motors propagate first through the individual filaments (that obey bending elasticity), and then through the branches and crosslinks (as well as other motors serving as crosslinks), possibly influencing the binding of other motors/linkers and the activity of other motors? It is well established that myosin acts cooperatively. Do such effects are taken by MEDYAN?
4. Can the authors address how would a more accurate study of rigidity percolation, e.g., the study of Broedersz, MacKintosh and coworkers, would affect the results?

To conclude, while the paper is interesting and could worthy publication, I do not recommend publication in its present form in PLOS Computational Biology. MEDYAN should be presented thoroughly, including Papoyan as a co-author, it should address the above questions, and include many more results showing the heterogeneities formed in the system. Otherwise, I think the paper does suit in its present form publication in a physical chemistry journal.
